# Change in Neighborhood Traffic Safety: Does It Matter in Terms of Physical Activity?

**DOI:** 10.1371/journal.pone.0062525

**Published:** 2013-05-02

**Authors:** Birthe Jongeneel-Grimen, Wim Busschers, Mariël Droomers, Hans A. M. van Oers, Karien Stronks, Anton E. Kunst

**Affiliations:** 1 Department of Public Health, Academic Medical Center (AMC), University of Amsterdam, Amsterdam, The Netherlands; 2 Centre for Public Health Status and Forecasting, National Institute for Public Health and the Environment, Bilthoven, The Netherlands; 3 Academic Collaborative Centre for Public Health Brabant, Tranzo, Faculty of Social Sciences, University of Tilburg, Tilburg, The Netherlands; McGill University, Canada

## Abstract

**Background:**

There is limited evidence on the causality of previously observed associations between neighborhood traffic safety and physical activity (PA). This study aims to contribute to this evidence by assessing the extent to which changes over time in neighborhood traffic safety were associated with PA.

**Methods:**

Data were accessed from the national survey Netherlands Housing Research for 2006 and 2009. The two samples of in total 57,092 Dutch residents aged 18–84 years lived in 320 neighbourhoods. Using multi-level hurdle models, the authors assessed whether the odds of being physically active and the mean hours of PA among active people (in 2009) were related to the levels of neighborhood traffic safety (in 2006) and changes in the levels of neighborhood traffic safety (between 2006 and 2009). Next, we examined if these associations varied according to gender, age, and employment status.

**Results:**

Higher levels of neighborhood traffic safety were associated with higher odds of being active (OR 1.080 (1.025–1.139)). An increase in levels of neighborhood traffic safety was associated with increased odds of being active (OR 1.060 (1.006–1.119)). This association was stronger among women, people aged 35 to 59, and those who were gainfully employed. Neither levels of traffic safety nor changes in these levels were associated with the mean hours of PA among people who were physically active (OR 0.997 (0.975–1.020); OR 1.001 (0.978–1.025), respectively).

**Conclusion:**

Not only levels of neighborhood traffic safety, but also increases in neighborhood traffic safety were related to increased odds of being active. This relationship supports claims for a causal relationship between neighborhood traffic safety and PA.

## Introduction

Regular physical activity (PA) is strongly related to better health outcomes [1]. Population levels of PA remain relatively low in most countries. In the Netherlands, 56% of the Dutch population over 15 years of age is not sufficiently physically active [2].

Social ecological models [3] posit that factors at multiple levels (including individual, social, and physical environmental factors) all influence health behaviors such as PA. A growing number of studies have reported neighborhood environmental factors that are associated with adults' PA. Examples of such factors are neighborhood safety, access to facilities, and enjoyable scenery [4].

Neighborhood traffic safety as a possible determinant of PA has received much attention in recent literature. Several cross-sectional studies have assessed the association between traffic safety or aspects of traffic safety (e.g., traffic volume, traffic speed) and PA [5]–[17], including walking [10], [17]–[25], among adults. The large majority of these studies used self-reported measures of PA [5]–[8], [10]–[21], [23]–[25]. A meta-analysis [26] reported mostly positive associations across studies between PA and the absence of traffic. Some other studies replicated this finding [6], [7], [13]. However, of the more recent studies, most reported that traffic safety was not associated with PA [5], [8]–[12], [15]–[17]. With regard to walking, recent research on the association with traffic safety showed inconsistent results. Six studies showed a lack of association [10], [19]–[23], two studies found a positive association [24], [25], and two studies reported a negative association [18], [22].

Virtually all studies measured traffic safety at one moment in time. Yet, in order to obtain stronger evidence on the causality of the reported associations, more advanced study designs are needed, including evaluations of “natural policy experiments” and studies that focus on more general changes in traffic safety [27]. Even so, almost no studies have assessed the effect of changes (as opposed to levels) in traffic safety on PA behavior. To our knowledge, the only exception to this was a study by Humpel et al. [28], which however measured changes over a period of only 10 weeks.

The present study aimed to investigate the extent to which levels of traffic safety and changes over time in levels of traffic safety were associated with participation in physical activity and sport among adults. The specific aims were:

- First, in an analysis of traffic safety measured at one moment in time, we aimed to assess whether traffic safety measured in 2006 was related to the odds of being physically active and the mean hours of PA (among people who were physically active) in 2009.
- Second, we focused on neighborhood changes in the levels of traffic safety between 2006 and 2009. We aimed to assess whether positive changes in levels of traffic safety were associated with increased odds of being active and with increased mean hours of PA in 2009.
- Finally, we aimed to assess whether these associations varied according to gender, age, and employment status.

For this last sub-aim, we expected to find the effect of traffic safety to be stronger for residents who spent a great deal of time in their neighborhood, who, in the Dutch context, are generally women, older people, and those who were not gainfully employed. A few studies have found the association between traffic safety and PA to be gender-specific [28]–[30]. Humpel et al. [28] demonstrated that increases in perceived traffic safety were related to an increase in walking in women but not in men. Timperio et al. [29] and Ishii et al. [30] reported that traffic safety was negatively associated with PA/active commuting among males, while no association was found among females. Older people might be particular vulnerable to unsafe traffic [31]. An USA study [32] found a negative association between neighborhood safety and inactivity for adults aged over 65 years, but not for younger adults.

## Methods

### Population

We used data from the cross-sectional Netherlands Housing Survey 2006 and 2009 (WoON06 and WoON09) [33], [34], conducted by The Ministry of Housing, Spatial Planning, and the Environment and the Central Bureau of Statistics. The WoON is a large-scale three-yearly national survey among people aged 18 and over. This survey focuses on housing quality and housing needs, but also includes data on both traffic safety and PA among respondents in various areas. Sample selection was conducted using municipal registration information. The samples drawn within municipalities were stratified by clusters defined in terms of age, gender, country of birth, and municipality (i.e. living or not in one of the four largest municipalities). The sample design took account that population groups with different demographic and geographic characteristics show differences in response-rates. Some municipalities were oversampled. The data collection was carried out by both telephone, face to face interviewing, and internet. The response rates were 70.9% and 62.6% for WoON06 and WoON09 respectively.

A neighborhood was defined by its 4-digit postal code. In the Netherlands, these areas are 8.3 km^2^ and comprise approximately 4,000 residents, on average. To accurately assess changes in neighborhood traffic safety between 2006 and 2009 we included only neighborhoods that had a minimum of 30 respondents in both surveys. As a result, we included in total 25,309 (WoON06) and 31,783 (WoON09) respondents living in 320 neighborhoods and aged 18 to 84.

### Outcome Variable

**Figure 1:**
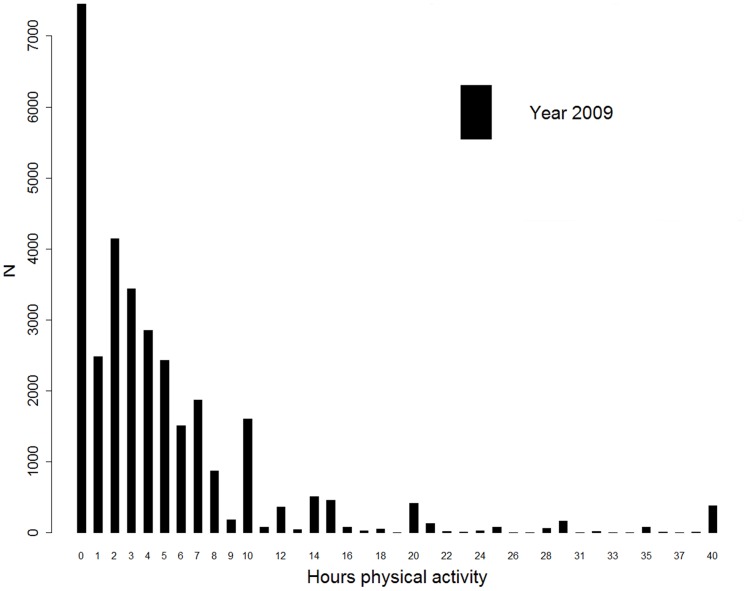
Frequency distribution of PA.

Our dependent variable was self-reported PA by 2009 respondents. PA was measured in the single question “How many hours a week do you spend on physical activity or sports?” Figure 1 presents the frequency distribution of the reported number of hours (range 0–40 hours). A large number of people reported 0 hours of PA (23.4%). For those who were engaging in PA at least one hour per week (referred to as physically active), the mean was 6.3 hours of PA. Respondents who reported more than 40 hours of PA per week, were assumed to be physically active for 40 hours per week.

### Predictor Variables

The main predictor variables in this study were the levels of traffic safety in 2006 and changes in these levels between 2006 and 2009. In the WoON surveys, traffic safety levels in 2006 and 2009 were measured using an identical statement “I think the traffic situation in this neighborhood is safe”, with responses on a 5-point scale varying from “strongly agree” to “strongly disagree.” The level of traffic safety in neighborhoods in 2006 and 2009 respectively was measured as the percentage of the neighborhood population who replied positively (“strongly agree” or “agree”) to this question.

The levels of traffic safety were thus measured at the neighborhood-level, by averaging the individual-level measures over all respondents living in the same postal code area, for 2006 and 2009 separately. In a similar way, changes in the levels of traffic safety between 2006 and 2009 were measured at the neighbourhood level, by subtracting each neighborhood's score in 2006 from that in 2009.

A range of sociodemographic covariates were used as control variables at the individual-level. These were gender (male and female), age (continuous variable), employment status (gainfully employed versus not gainfully employed), education (four categories, based on the highest educational level achieved: no education/elementary, lower secondary, upper secondary, and tertiary education), and disposable equivalent household income (categorized in quartiles, calculated by dividing the disposable household income by the square root of the number of household members) [35].

### Data Analyses

First, we used scatter plots to examine the association of PA with the levels of neighborhood traffic safety in 2006 and changes therein between 2006 and 2009. In agreement with subsequent analyses (see below), PA was decomposed in two measures: the proportion of respondents who reported at least one hour of PA, and number of hours of PA for the physically active respondents. The first measure was presented as odds by plotting it on the logit scale, while the latter measure was presented on the logarithmic scale.

Hurdle models were used to investigate how PA outcomes among the 2009 survey respondents were related to neighborhood and individual characteristics. Hurdle models apply a two-component approach, which combines the analysis of factors associated with the prevalence of PA (i.e. the odds of being physically active; the first component) and factors associated with the frequency of PA (i.e. the mean hours of PA among those who were physically active (at least one hour per week); the second component). Hurdle models have the advantage of being able to model the disproportionate number of respondents with 0 hours of PA (see Figure 1). Furthermore, they allow for separate estimates of the factors associated with prevalence of activity and with the amount of activity.

Our model was estimated using logistic regression to model the dichotomous outcome of being physically active, while a zero-truncated negative binomial regression was used to model the mean hours of PA among those who were physically active. For the logistic regression analyses, the association with predictors is reported using odds ratios (ORs) and 95% confidence intervals (CIs). For the negative binomial regression analyses, associations are reported using activity intensity ratios and 95% CIs. The activity intensity ratio is interpreted as the decrease or increase in the number of hours of PA per one-unit increase in the independent variable.

For the regression analyses, we rescaled the traffic safety variable in such a way that one unit increase corresponds to a meaningful change in levels of traffic safety within neighborhoods, which we defined as a 10 % increase in the number of residents who report their neighborhood to be safe. Hence, an odds ratio of 1.20 indicates an increase of 20% in the odds of PA when neighborhood safety levels increase by this 10%, while activity intensity ratio of 1.20 indicates an increase of 20% in the number of hours of PA.

All regression models included gender, age, employment status, education, and household income as individual-level control variables. To account for possible dependencies between observations within a neighborhood, we included random intercepts in the model. Level 1 was defined as the individual, and level 2 was defined as neighborhood.

We included two area-level variables: level of neighborhood traffic safety in 2006, and degree of change in traffic safety between 2006 and 2009. In a next step, we decomposed the change variable into two components: a measure identifying areas with improvement in traffic safety (equal to the change variable if change was positive; 0 if change was negative) and a measure identifying areas with decline in traffic safety (equal to the change variable if change was negative; 0 if change was positive). Finally, we applied stratified analyses to assess whether associations varied according to gender, age, and employment status.

Data were analyzed using the statistical packages R version 2.11.1 and SAS version 9.2.

## Results

Table 1 shows that, on average, there were small differences between the survey participants in 2006 and 2009 with regard to distribution by gender, age, educational level, and household income. About 10% more 2006 survey participants were not gainfully employed compared to the 2009 survey sample.

**Table 1:** Descriptive statistics of the study sample for 2006 (*N* = 25,309) and 2009 (*N* = 31,783) respectively.

	Respondents in 2006 (%)	Respondents in 2009 (%)
Gender*		
Female	53.42	55.61
Age (in years)*		
18–34	29.27	28.74
35–59	43.74	42.94
60–84	26.99	28.32
*Socioeconomic variables*		
Employment status*		
Not gainfully employed	42.38	32.92
Educational level*		
No education/elementary	12.32	8.72
Lower secondary	30.78	28.13
Upper secondary	32.16	35.40
Tertiary education	24.73	27.74
Disposable equivalent household income		
Low	25.74	26.25
Medium-low	25.03	25.29
Medium-high	24.88	25.03
High	24.35	23.42

* The distribution in 2009 is different from the distribution in 2006 with P≤0.05, two-sided. Generalized linear mixed models, corrected for neighborhood-level clustering effects.

Table 2 shows that on average 68% of the neighborhood population found the traffic situation in their neighborhood to be safe. This neighborhood traffic safety score varied from approximately 58% for the neighborhoods at the 10^th^ percentile to about 79% for the neighborhoods at the 90^th^ percentile.

**Table 2:** Mean, standard deviations (SD), and percentile distribution for traffic safety in 2006 and change in traffic safety between 2006 and 2009, for 2009 respondents.

	Predictors	
	Traffic Safety^a^	Change in Traffic Safety^b^
Mean	68.22	−4.47
SD	8.99	8.28
Percentiles of respondents (Percentiles of neighborhoods)		
10	57.69 (57.80)	−14.34 (−16.69)
25	63.83 (63.58)	−9.81 (−9.84)
50	68.57 (69.23)	−4.33 (−3.64)
75	74.58 (75.00)	1.05 (1.79)
90	78.99 (79.42)	5.13 (7.72)

a Measured as the % of the neighborhood population in 2006 who think the traffic situation in this neighborhood is safe.

b Measured by subtracting the traffic safety neighborhood score in 2006 from that in 2009, per neighborhood.

**Figure 2:**
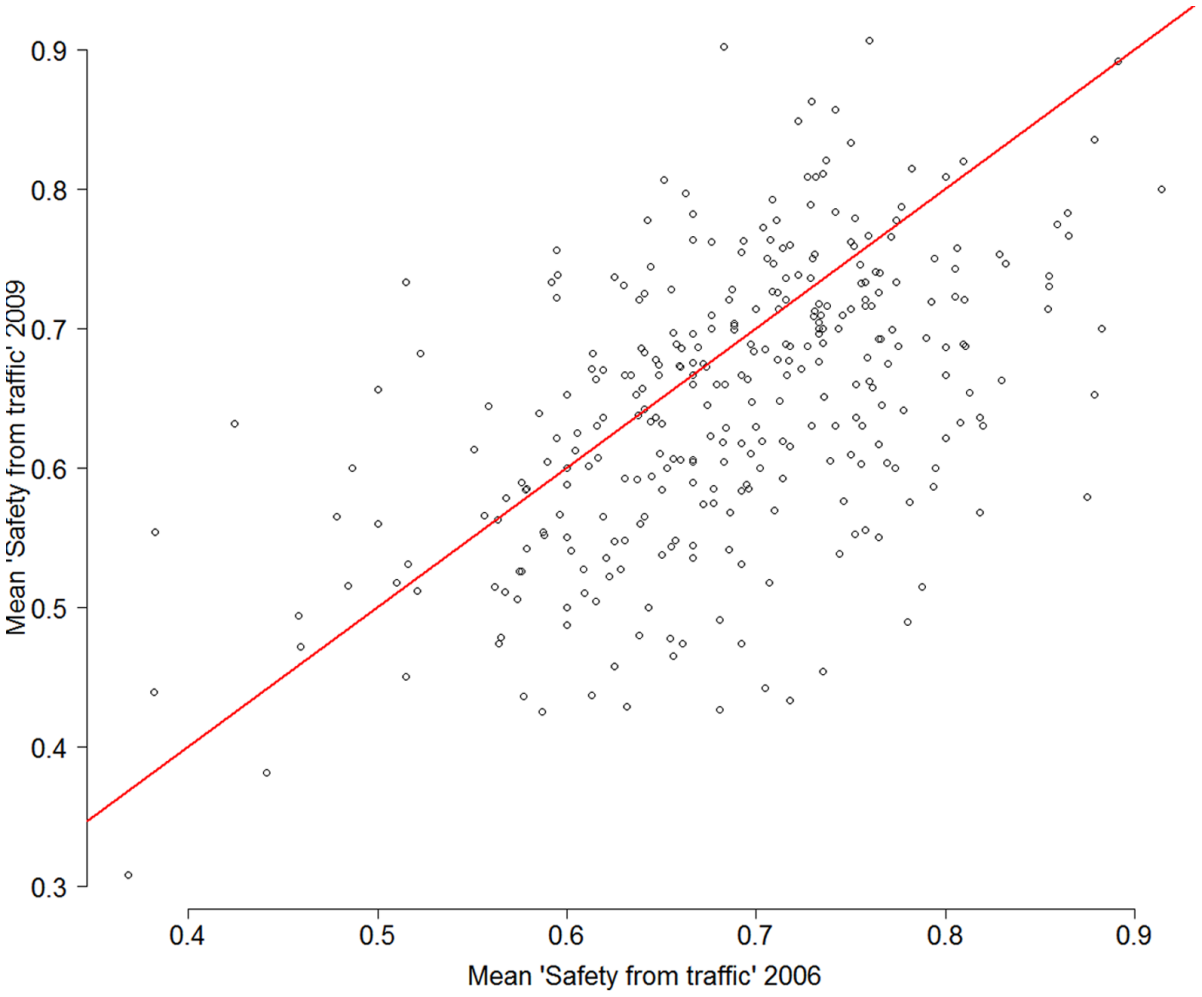
Traffic safety in neighborhoods: 2009 levels plotted against 2006 levels (both measured at neighborhood level). Correlation coefficient is 0.52.

Figure 2 visualizes the levels of neighborhood traffic safety scores in the years 2006 and 2009. Levels of neighborhood traffic safety scores of the survey samples in 2006 and 2009 were moderately correlated (a correlation coefficient of 0.52). Many neighborhoods experienced either a main improvement or a main deterioration in reported levels of traffic safety between 2006 and 2009.

**Figure 3:**
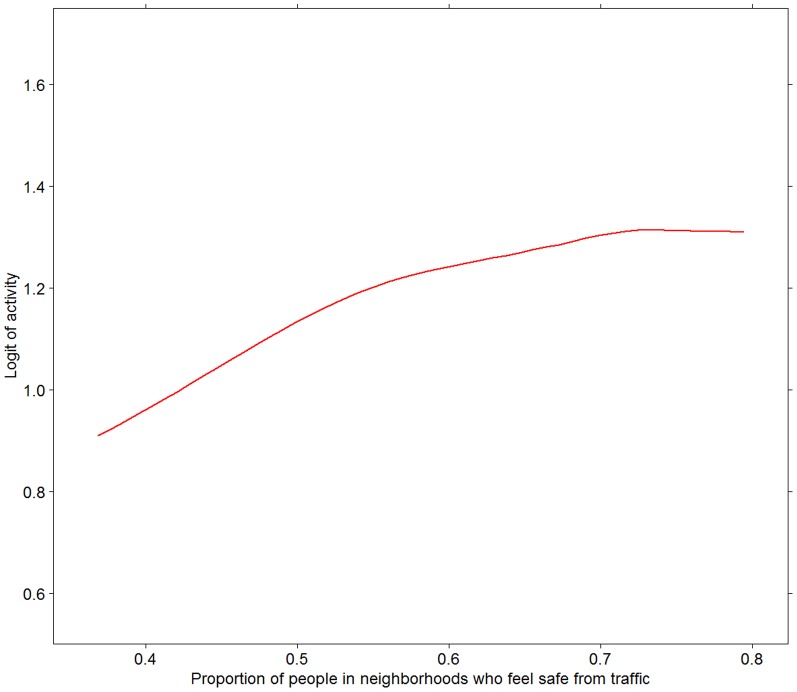
Prevalence of PA in 2009 in relation to levels of traffic safety in 2006. Prevalence of PA is measured as crude logit. Relationship depicted by a Loess curve based on 1 degree of freedom.

**Figure 4:**
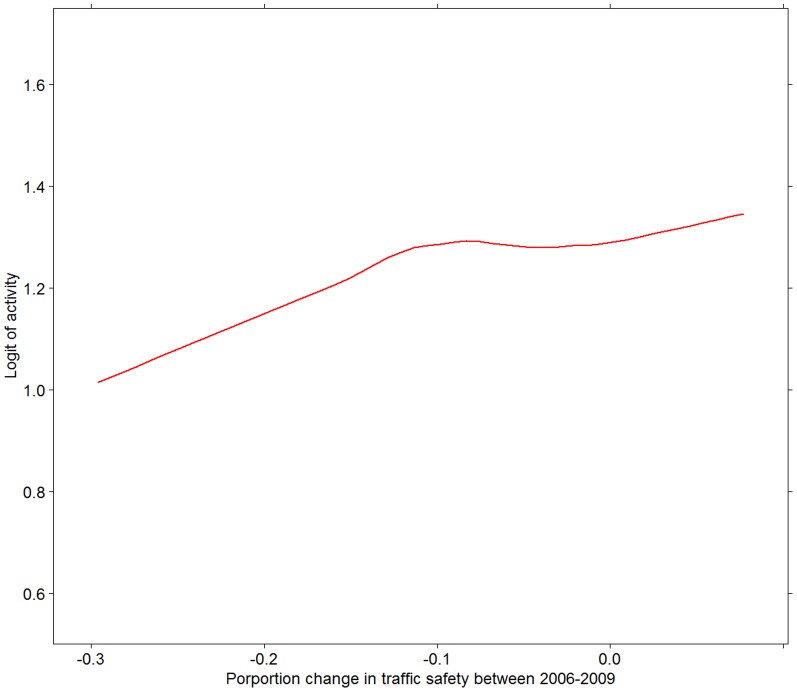
Prevalence of PA in 2009 in relation to change in traffic safety between 2006 and 2009. Prevalence of PA is measured as crude logit. Relationship depicted by a Loess curve based on 1 degree of freedom.

**Figure 5:**
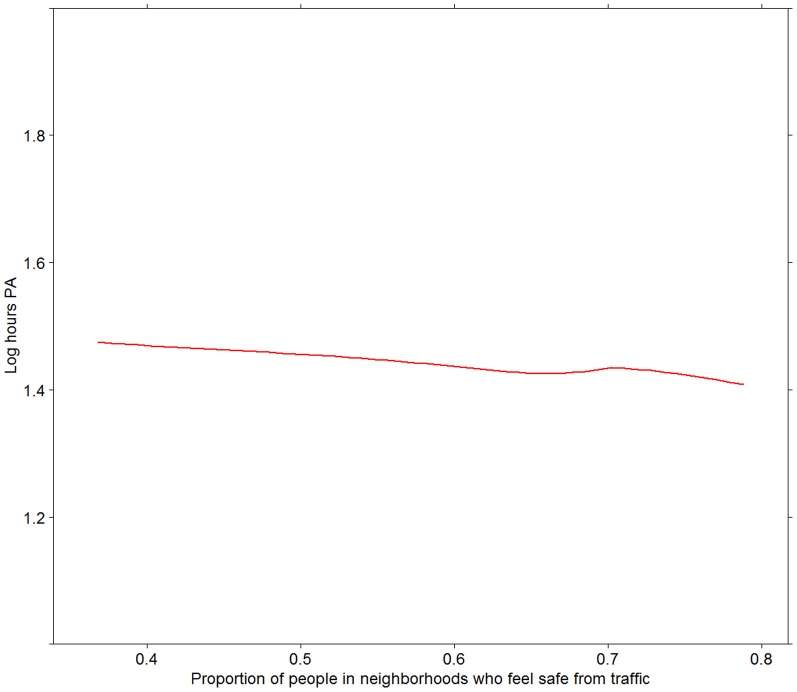
Frequency of PA in relation to levels of traffic safety in 2006. Frequency of PA is measured as log of hours of PA among those who are active. Relationship depicted by a Loess curve based on 1 degree of freedom.

**Figure 6:**
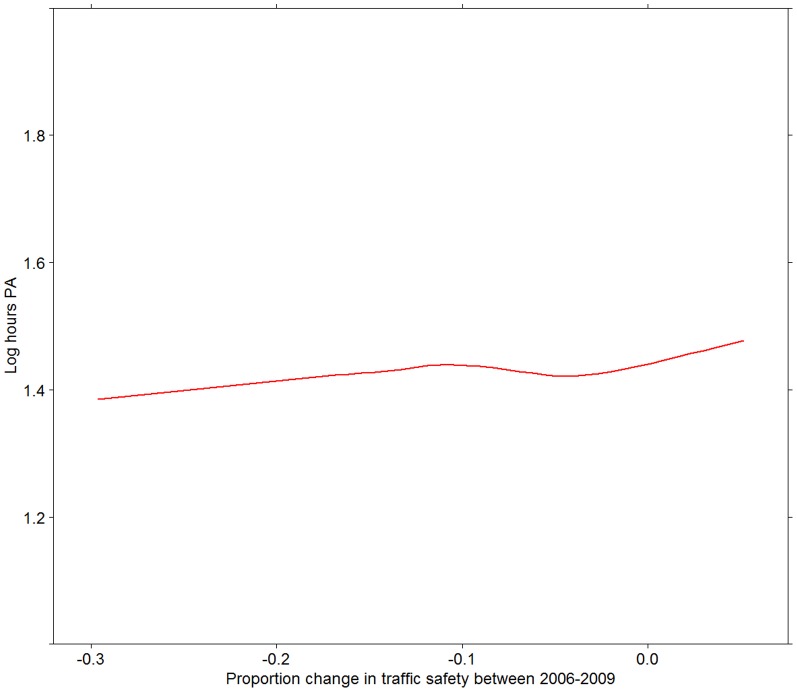
Frequency of PA in relation to change in traffic safety between 2006 and 2009. Frequency of PA is measured as log of hours of PA among those who are active. Relationship depicted by a Loess curve based on 1 degree of freedom.

Figures 3, 4, 5, 6 present associations of the two traffic safety measures (both levels in 2006 and changes between 2006 and 2009) with the two PA variables. The proportion of people reporting PA in 2009 was positively associated with both the levels of traffic safety in 2006 and changes in the levels of traffic safety between 2006 and 2009. No associations were found between the mean hours of PA (among those who were physically active) and the two traffic safety variables (Figures 5 and 6).

Table 3 shows that living in neighborhoods characterized by higher levels of traffic safety was associated with increased odds of being active. An increase in the levels of traffic safety between 2006 and 2009 was associated with an increase in odds of the neighborhood population being active. Neither the levels of traffic safety in 2006 nor the trends in the period 2006–2009 were associated with the mean hours of PA (among those who were physically active).

**Table 3:** Association of physical activity in 2009 with traffic safety in 2006 and change in traffic safety between 2006 and 2009, and sociodemographic variables, for 2009 respondents^a^.

	Prevalence of Being Physically Active in 2009 (zero^b^ versus at least 1 hour per week)	Frequency of Physical Activity in 2009 (number of hours of PA, among those who are active)
Predictors	Odds ratios (95% CI)	Activity Intensity Ratio^c^ (95% CI)
Neighborhood		
Traffic safety in 2006^d^	1.080 (1.025–1.139)*	0.997 (0.975–1.020)
Change in traffic safety in 2006–2009^e^	1.060 (1.006–1.119)*	1.001 (0.978–1.025)
Individual		
Age (in years)	0.995 (0.993–0.997)*	1.003 (1.002–1.004)*
Gender		
Male	1.000	1.000
Female	1.036 (0.980–1.095)	0.797 (0.775–0.819)*
Education		
No education/elementary	1.000	1.000
Lower secondary	1.642(1.499–1.799)*	1.031 (0.971–1.094)
Upper secondary	2.551 (2.315–2.801)*	0.911 (0.858–0.967)*
Tertiary	4.016 (3.610–4.464)*	0.776 (0.730–0.825)*
Employment status		
Not gainfully employed	1.000	1.000
Gainfully employed	0.858 (0.795–0.925)*	0.918 (0.883–0.954)*
Household income		
Low	1.000	1.000
Medium-low	1.344 (1.248–1.445)*	0.984 (0.945–1.025)
Medium-high	1.555 (1.437–1.684)*	0.928 (0.891–0.967)*
High	1.953 (1.786–2.132)*	0.917 (0.879–0.958)*

* The association is statistically significant by P≤0.05, two-sided.

a The analysis only includes neighborhoods with a minimum of 30 respondents per survey year.

b Reference group.

c Activity intensity ratio for those who reported being physically active for at least one hour per week.

d One unit change corresponds to an increase by 10% points in the proportion of residents reporting their neighbourhood to be safe.

e One unit change corresponds to an increase by 10% points in changes in levels of traffic safety between 2006 and 2009.

Table 4 shows that the odds of the neighborhood population being active in 2009 were slightly higher in districts that experienced a positive change in traffic safety between 2006 and 2009, and slightly lower in districts with a negative change (though the latter associations were not statistically significant). Neither a positive or negative change in the levels of traffic safety between 2006 and 2009 were associated with the mean hours of PA (among those who were physically active).

**Table 4:** Association of physical activity in 2009 with traffic safety in 2006 and the degree of positive or negative change in traffic safety between 2006 and 2009, for 2009 respondents^a^.

	Prevalence of Being Physically Active in 2009 (zero^b^ versus at least 1 hour per week)	Frequency of Physical Activity in 2009 (number of hours of PA, among those who are active)
Predictors^d^	Odds ratios (95% CI)	Activity Intensity Ratio^c^ (95% CI)
Neighborhood		
Traffic safety in 2006^e^	1.087 (1.031–1.147)*	1.002 (0.979–1.026)
Positive change in traffic safety in 2006–2009^f^	1.071 (0.943–1.215)	0.998 (0.943–1.056)
Negative change in traffic safety in 2006–2009^f^	1.065 (0.989–1.147)	0.997 (0.964–1.030)

* The association is statistically significant by P≤0.05, two-sided.

a The analysis only includes neighborhoods with a minimum of 30 respondents per survey year.

b Reference group.

c Activity intensity ratio for those who reported being physically active for at least one hour per week.

d Controlled for gender, age, employment status, education, and household income.

e One unit change corresponds to an increase by 10% points in the proportion of residents reporting their neighbourhood to be safe.

f One unit change corresponds to an increase by 10% points in changes in levels of traffic safety between 2006 and 2009.

Table 5 shows that, for all demographic subgroups, the levels of traffic safety in 2006 and the trends in the period 2006–2009 were consistently associated with increased odds of the neighborhood population being active. These associations were stronger among women, people aged 35 to 59, and those who were gainfully employed. The association with levels of traffic safety in 2006 was also relatively strong among people aged 18 to 34. As in previous analyses, no associations were observed with the mean hours of PA (among those who were physically active).

**Table 5:** Association of physical activity in 2009 with traffic safety in 2006 and change in traffic safety between 2006 and 2009 according to gender, age, and employment status, for 2009 respondents^a^.

	Predictors			
	Traffic Safety in 2006^b^		Change in Traffic Safety in 2006–2009^c^	
	Prevalence of Being Physically Active in 2009^de^	Frequency of Physical Activity in 2009^f^	Prevalence of Being Physically Active in 2009^de^	Frequency of Physical Activity in 2009^f^
	Odds ratios (95% CI)	Activity Intensity Ratio^g^ (95% CI)	Odds ratios (95% CI)	Activity Intensity Ratio^g^ (95% CI)
Gender^h^				
Male	1.055 (0.991–1.124)	0.998 (0.968–1.029)	1.050 (0.985–1.121)	0.999 (0.968–1.031)
Female	1.096 (1.031–1.166)*	1.002 (0.971–1.033)	1.066 (1.002–1.135)*	1.006 (0.974–1.038)
Age^h^ (in years)				
18–34	1.085 (1.004–1.171)*	0.997 (0.961–1.036)	1.014 (0.935–1.100)	0.995 (0.956–1.035)
35–59	1.109 (1.037–1.185)*	0.989 (0.956–1.023)	1.078 (1.008–1.153)*	0.999 (0.965–1.034)
60–84	1.028 (0.953–1.107)	1.017 (0.983–1.052)	1.042 (0.964–1.125)	1.013 (0.978–1.049)
Employment status^h^				
Gainfully employed	1.099 (1.035–1.167)*	1.003 (0.976–1.031)	1.063 (1.000–1.129)*	1.006 (0.979–1.034)
Not gainfully employed	1.055 (0.981–1.134)	0.983 (0.951–1.016)	1.052 (0.976–1.133)	0.986 (0.952–1.020)

* The association is statistically significant by P≤0.05, two-sided.

a The analysis only includes neighborhoods with a minimum of 30 respondents per survey year.

b One unit change corresponds to an increase by 10% points in the proportion of residents reporting their neighbourhood to be safe.

c One unit change corresponds to an increase by 10% points in changes in levels of traffic safety between 2006 and 2009.

d Odds ratios were calculated on zero versus at least 1 hour physically active per week.

e Reference group were respondents who reported zero hours of PA.

f Number of hours of PA, among those who reported being physically active for at least one hour per week.

g Activity intensity ratio for those who reported being physically active for at least one hour per week.

h Control variables include gender, age, employment status, education, and household income (when applicable).

## Discussion

### Key Findings

This study explores the extent to which the levels of traffic safety in 2006 and changes over time in the levels of traffic safety were associated with PA. We found that levels of neighborhood traffic safety in 2006 were related to increased odds of the neighborhood population being active in 2009. However, traffic safety levels were not related to the mean hours of PA (among those who were physically active). Similarly, we found that an increase in traffic safety levels between 2006 and 2009 was related to increased odds of being active in 2009, but not to the mean hours of PA. Thirdly, these positive associations with the odds of being active were found for all demographic groups, but tended to be stronger among women, people aged 35 to 59, and those who were gainfully employed.

### Evaluation of Potential Data Problems

Selective migration is a potential bias if individuals who are already physically active select neighborhoods based on their activity-related amenities [36]. Cross-sectional studies with a one-time measure of environmental conditions are particularly vulnerable to such bias. However, in our study, we focused on changes in neighborhood traffic safety over a relatively short time span. As migration flows are unlikely to respond rapidly and substantially within such a short period of time, we expect selective migration to have a negligible effect on the associations that we observed with changes in traffic safety.

The response rates were 70.9% and 62.6% for WoON06 and WoON09 respectively. These non-response rates do not count people who were unapproachable because of recent death, recent emigration, or untraceable addresses. If the non-response had been strongly related to both neighborhood traffic safety and PA, this would have biased our results. The WoON06 and WoON09 surveys show that non-response rates vary little according to place of residence [33], [34], suggesting a weak relationship to levels of neighborhood traffic safety. Though non-response bias cannot be excluded, we expect this effect to be minor.

Traffic safety was measured with only one broad question, which aimed to cover several aspects of local safety. This might have been an advantage compared to studies that measured only one aspect of traffic safety, such as traffic volume [8], [10], [14], [22]. Nonetheless, the ideal would be to measure traffic safety using a reliable and validated questionnaire consisting of multiple items that cover different aspects of traffic safety [7]. Future research should assess changes over time in levels of traffic safety using more comprehensive indicators of traffic safety.

Research has shown the relation between subjective and objective traffic safety to be weak [37]. In this paper, we preferred to measure traffic safety with self-reports rather than with objective measures like for example the registered number of accidents. Reports of traffic safety may reflect feelings of being safe in traffic as experienced by the residents [37]. People's decision to be active or not in the neighborhood will probably depend more directly on these personal experiences of safety than on objective traffic safety. Given our aim to asses association of changes in traffic safety with PA, the use of self-report measures may be more adequate. If, on the other hand, our aim would have been to assess which areas were most in need for traffic safety interventions, objective measures would be preferred.

The population surveys used in this study only provided one single question to assess PA. Ideally, we would have used detailed self-reports of PA assessment [38] or objective measurement devices such as pedometers and accelerometers. Self-reports of PA suffers from substantial reporting bias [39] due to both social desirability bias and the cognitive challenge to accurately recall frequency and duration of PA [40]. For example, studies have shown that adherence to PA recommendations according to self-report is substantially higher than according to objectively measured activity [41]. The use of self-reports could have led to an overestimation of PA, even though the association with environmental safety may not be biased. Future research should try to replicate our findings from this study using objective measurements of PA.

Our PA measure asked respondents about engagement in physical activity or sports in general, part of which would have taken place outside the neighborhood. However, PA also includes walking and bicycling, which in the Netherlands are highly prevalent [42]. Furthermore, as shops and facilities are within acceptable walking and cycling limits for much of the Dutch population, much of walking and cycling occurs within or close to the neighbourhood [43]. Nonetheless, if the associations observed in our study are truly causal, it seems likely that these relationships would have been stronger with measures focusing on neighborhood-related PA.

Levels of traffic safety were measured at the level of neighborhoods by aggregating information from all respondents living in these same neighborhoods. To obtain sufficiently stable estimates, we restricted the analyses to neighborhoods with a least 30 respondents in both surveys. In further analyses, we evaluated whether we would have obtained other results by further restricting the analyses to neighborhoods with a minimum of 50 respondents in both surveys (Table 6). Among this more restrictive set of areas, we also observed the positive associations reported above. However, partly due to the lower number of areas and respondents (40% less than in the main analyses), the associations were weaker and not statistically significant.

**Table 6:** Association of physical activity in 2009 with traffic safety in 2006 and change in traffic safety between 2006 and 2009, and sociodemographic variables, for 2009 respondents^a^.

	Prevalence of Being Physically Active in 2009 (zero^b^ versus at least 1 hour per week)	Frequency of Physical Activity in 2009 (number of hours of PA, among those who are active)
Predictors	Odds ratios (95% CI)	Activity Intensity Ratio^c^ (95% CI)
Neighborhood		
Traffic safety in 2006^d^	1.048 (0.962–0.143)	1.001 (0.967–1.035)
Change in traffic safety in 2006–2009^e^	1.034 (0.949–1.129)	0.984 (0.951–1.019)
Individual		
Age (in years)	0.993 (0.991–0.996)*	1.003 (1.002–1.004)*
Gender		
Male	1.000	1.000
Female	1.050 (0.979–1.127)	0.791 (0.765–0.819)*
Education		
No education/elementary	1.000	1.000
Lower secondary	1.629 (1.449–1.832)*	1.029 (0.954–1.110)
Upper secondary	2.532 (2.237–2.857)*	0.904 (0.838–0.975)*
Tertiary	3.759 (3.279–4.310)*	0.770 (0.712-0.833)*
Employment status		
Not gainfully employed	1.000	1.000
Gainfully employed	0.776 (0.704–0.855)*	0.927 (0.883–0.974)*
Household income		
Low	1.000	1.000
Medium-low	1.416 (1.290–1.555)*	0.988 (0.938–1.040)
Medium-high	1.647 (1.490–1.821)*	0.939 (0.891–0.989)*
High	2.165 (1.931–2.421)*	0.934 (0.884–0.986)*

* The association is statistically significant by P≤0.05, two-sided.

a Sensitivity analysis with 50 respondents per neighborhood.

b Reference group.

c Activity intensity ratio for those who reported being physically active for at least one hour per week.

d One unit change corresponds to an increase by 10% points in the proportion of residents reporting their neighbourhood to be safe.

e One unit change corresponds to an increase by 10% points in changes in levels of traffic safety between 2006 and 2009.

If sociodemographic differences exist between the 2006 and 2009 survey sample by neighborhood, then the differences in traffic safety could not represent real changes in traffic safety but merely represent a function of differences in respondents' composition. Furthermore, if such differences were correlated with PA this may have led to an overestimation of the association between traffic safety and PA. To measure changes in traffic safety more accurately, future studies should use longitudinal designs or objective measures of the environment.

### Explanations

A recent Dutch study among children found that higher perceived traffic safety in neighborhoods was associated with more PA [44]. It was suggested that traffic safety may influence PA because parents discourage their children from playing outside in their neighborhood when they perceive local traffic to be unsafe. If the same reasoning applies to the adults themselves, they would restrict PA in their local environment if they perceive traffic to be unsafe. Other explanations may relate to problems closely related to traffic safety. For example, neighborhoods with low traffic safety and high traffic volume may have more air pollution and odor nuisance, which may in turn discourage local PA [45].

Our finding that the levels of traffic safety in 2006 were not associated with mean hours of PA is consistent with what has been found in most other studies that also looked at traffic safety and frequency of PA [5], [11], [16]. This suggests that traffic safety, if it has a causal effect on PA, it is not mainly because it would make active people become more active. The amount of time residents are physically active may be influenced more by psychosocial factors than by environmental factors [11]. However, we cannot exclude the possibility that the nonexistent association between traffic safety (both levels and changes) and mean hours of PA could possibly be explained by the relatively imprecise measurement used to assess the amount of PA among those who are active.

We had expected the effects to be stronger for older people and those not gainfully employed, as these people spend more time in their neighborhoods. However, the results did not support this expectation. One possible explanation may be that these groups include many people who are physically or mentally disabled. Future studies on the association between environmental factors and PA are recommended to specifically look at people with disabilities.

### Implications

Cross-sectional studies with a measure of traffic safety at one moment in time predominate the existing research on the effects on PA. We used a new study design that measured the effect of changes over time in the level of traffic safety and compared neighborhoods with different degrees of change. The associations observed with these change measures provide new and stronger evidence for a causal relationship between neighborhood traffic safety and PA. This new evidence supports the expectation that improving traffic safety in neighborhoods may result in an increase in PA among neighborhood residents. Even though the effect of improving traffic safety on individual-level PA may seem small, such improvements affect large populations over long periods of time. Future research should aim to add to this type of evidence by using the same design in different countries and contexts, or by using even stronger designs, such as longitudinal evaluations of natural policy experiments.
